# Supplementary material for: The association of developmental trajectories of adolescent mental health with early-adult functioning
Source: PLoS One. 2020 Jun 10;15(6):e0233648. doi: 10.1371/journal.pone.0233648 (PMC7286481; doi:10.1371/journal.pone.0233648)
Supplement: S1 Table — The decreasing-low INT+EXT class (n = 189) was used as a reference. OR = odds ratio, 95% CI = 95% confidence interval, INT = internalizing, EXT = externalizing, Unadj. = unadjusted model, Adj. = adjusted model (covariates were: childhood functioning, sex, IQ, temperament (effortful control, fear, frustration), family SES, parenting stress, perceived parenting (warmth and rejection), and current mental health). Covariates printed in italic had a protective effect as indexed by an OR<1. aadjusted analyses controlled for all covariates except childhood functioning data. badjusted analyses controlled for all covariates except current mental health. (PDF) [file pone.0233648.s001.pdf]

Table 4. Unadjusted and adjusted associations between the adolescent mental health trajectory classes (derived from the 6-class model) and early-adult functioning outcomes. The decreasing-low INT+EXT class (n=189) was used as a reference.

	**Continuous high INT+EXT (n=221) vs. reference**		**Decreasing, high EXT/moderate INT (n=265) vs. reference**		**Continuous moderate INT>EXT (n=303) vs. reference**		**Decreasing, moderate EXT/low INT (n=368) vs. reference**	
	**Unadj.**	**Adj.**	**Unadj.**	**Unadj.**	**Unadj.**	**Adj.**	**Unadj.**	**Adj.**
**Domain**	OR (95% CI)	OR (95% CI)	OR (95% CI)	OR (95% CI)	OR (95% CI)	OR (95% CI)	OR (95% CI)	OR (95% CI)
**Economic**								
Low educational level	4.25 (1.41-12.84)	4.15 (1.21-14.25)	3.10 (1.02-9.46)	1.18 (0.52-5.86)	1.91 (0.61-6.04)	1.81 (0.51-6.39)	1.61 (0.52-5.04)	3.31 (0.96-11.38)
Frequent absenteeism	2.87 (1.18-6.94)	2.44 (0.92-6.43)	1.51 (0.59-3.82)	1.51 (0.56-4.07)	1.37 (0.55-3.44)	1.33 (0.51-3.44)	0.84 (0.33-2.19)	0.96 (0.36-2.54)
Receiving social security benefits[a]	3.19 (1.25-8.15)	1.81 (0.62-5.31)	2.04 (0.78-5.34)	1.42 (0.48-4.18)	2.18 (0.86-5.56)	2.30 (082-6.41)	0.57 (0.19-1.71)	0.44 (0.13-1.45)
Serious financial difficulties[a]	14.47 (3.39-61.82)	14.66 (3.25-66.04)	7.58 (1.74-33.05)	8.53 (1.89-38.47)	2.90 (0.62-13.60)	3.22 (0.67-15.46)	1.49 (0.30-7.45)	1.63 (0.32-8.31)
**Social**								
Early parenthood[a]	2.99 (1.16-7.68)	2.44 (.087-6.84)	2.18 (0.84-5.66)	2.44 (0.88-6.72)	0.83 (0.28-2.44)	0.71 (0.23-2.15)	0.57 (0.19-1.71)	0.68 (0.22-2.11)
Multiple social difficulties[a]	10.33 (4.29-24.85)	9.84 (3.90-24.84)	4.60 (1.88-11.29)	5.25 (2.07-13.32)	4.36 (1.79-10.58)	4.67 (1.88-11.64)	2.90 (1.19-7.07)	3.58 (1.44-8.90)
Delinquency	7.14 (2.72-18.77)	8.49 (2.98-24.25)	4.96 (1.88-13.11)	4.53 (1.62-12.66)	2.05 (0.74-5.72)	3.53 (1.22-10.25)	4.97 (1.93-12.79)	*4.24 (1.60-11.21)*
**Health**								
Smoking	6.37 (2.77-14.67)	6.41 (2.65-15.50)	4.68 (2.03-10.78)	4.56 (1.91-10.85)	1.76 (0.72-4.29)	1.94 (0.78-4.82)	2.65 (1.15-6.11)	*2.69 (1.16-6.29)*

Problematic substance use	Alcohol	2.09 (0.83-5.21)	1.73 (0.63-4.77)	2.71 (1.13-6.46)	1.74 (0.68-4.48)	1.15 (0.45-2.96)	1.56 (0.58-4.20)	2.53 (1.10-5.85)	*1.55 (0.65-3.71)*
	Cannabis	5.84 (2.20-15.50)	10.07 (3.44-29.48)	3.97 (1.49-10.63)	5.14 (1.90-14.71)	0.61 (0.17-2.13)	1.13 (0.31-4.14)	2.88 (1.09-7.61)	2.58 (0.94-7.06)
Suicidality		8.66 (1.98-37.96)	8.00 (1.62-39.39)	0.37 (0.03-4.06)	0.35 (0.03-4.11)	2.56 (0.54-12.20)	2.72 (0.53-13.96)	0.24 (0.02-2.71)	0.25 (0.02-2.83)
Mental health concerns[b]	Unhappy /dissatis-faction	11.77 (4.11-33.71)	13.18 (4.32-40.24)	3.92 (1.31-11.70)	4.61 (1.48-14.36)	5.88 (2.04-16.92)	6.40 (2.15-19.02)	2.80 (0.95-8.27)	3.12 (1.03-9.45)
	Poor sleep quality	13.17 (3.07-56.50)	13.69 (3.02-62.12)	4.22 (0.92-19.30)	4.78 (1.01-22.70)	10.84 (2.56-45.96)	11.77 (2.70-51.29)	2.50 (0.54-11.55)	2.81 (0.60-13.19)
	Loneliness	24.32 (3.25-182.06)	37.21 (4.71-293.89)	10.08 (1.30-77.92)	16.10 (2.02-128.60)	14.93 (1.99-111.93)	19.69 (2.56-151.55)	0.97 (0.09-10.83)	1.15 (0.10-12.99)
Serious physical event[a]		4.10 (0.87-19.27)	3.71 (0.72-19.12)	3.81 (0.82-17.62)	4.20 (0.86-20.50)	2.21 (0.45-10.79)	2.21 (0.44-11.17)	1.73 (0.36-8.44)	1.86 (0.37-9.23)
Obesity		2.64 (1.08-6.42)	1.48 (0.55-3.98)	2.04 (0.84-4.99)	1.59 (0.60-4.17)	1.44 (0.58-3.59)	1.04 (0.40-2.75)	1.26 (0.52-3.10)	1.28 (0.50-3.26)
Poor subjective physical health		14.23 (5.52-36.64)	14.38 (5.37-38.47)	5.67 (2.16-14.88)	6.70 (2.49-18.03)	8.91 (3.49-22.79)	8.54 (3.29-22.20)	3.44 (1.32-9.00)	4.10 (1.55-10.86)

Note. OR=odds ratio, 95% CI=95% confidence interval, INT=internalizing, EXT=externalizing, Unadj.=unadjusted model, Adj.=adjusted model (covariates were: childhood functioning, sex, IQ, temperament (effortful control, fear, frustration), family SES, parenting stress, perceived parenting (warmth and rejection), and current mental health).

Covariates printed in *italic* had a protective effect as indexed by an OR<1

[a]adjusted analyses controlled for all covariates except childhood functioning data

[b]adjusted analyses controlled for all covariates except current mental health

Table 4. Continued

	**Increasing, EXT>INT (n=178) vs. reference**		**Explained variance (R^2)**		**Estimates (OR; 95% CI) for INT and EXT**
	**Unadj.**	**Adj.**	Unadj.	Adj.	Adj.
**Domain**	OR (95% CI)	OR (95% CI)			
**Economic**					
Low educational level	4.23 (1.36-13.17)	4.14 (1.23-13.92)	.03	.30	INT: 6.47 (1.86-22.54) EXT: 1.93 (0.52-7.18)
Frequent absenteeism	0.63 (0.18-2.20)	0.73 (0.21-2.61)	.03	.09	INT: 2.85 (0.89-9.17) EXT: 2.30 (0.60-8.82)
Receiving social security benefits[a]	1.33 (0.44-4.04)	1.07 (0.32-3.55)	.05	.29	INT: 7.65 (2.51-23.38) EXT: 6.27 (1.84-21.35)
Serious financial difficulties[a]	7.28 (1.60-33.11)	7.54 (1.62-35.01)	.11	.17	INT: 2.21 (0.64-6.40) EXT: 4.83 (1.44-16.20)
**Social**					
Early parenthood[a]	1.33 (0.44-4.04)	1.57 (0.49-4.99)	.05	.17	INT: 0.79 (0.20-3.14) EXT: 4.69 (1.10-19.99)
Multiple social difficulties[a]	5.49 (2.18-13.84)	6.43 (2.49-16.65)	.07	.16	INT: 1.03 (0.43-2.47) EXT: 10.74 (4.13-27.93)
Delinquency	7.54 (2.82-20.14)	6.37 (2.30-17.59)	.06	*.19*	INT: 0.22 (0.07-0.68) EXT: 21.32 (7.34-61.92)
**Health**					
Smoking	4.67 (1.95-11.17)	4.29 (1.77-10.43)	.06	*.12*	INT: 0.26 (0.09-0.74) EXT: 5.37 (1.97-14.66)

Problematic substance use	Alcohol	3.51 (1.43-8.57)	2.32 (0.91-5.91)	.03	*.13*	INT: 0.21 (0.05-0.86) EXT: 3.90 (1.19-12.78)
	Cannabis	7.42 (2.78-19.82)	7.34 (2.63-20.49)	.09	.21	INT: 1.07 (0.34-3.32) EXT: 5.28 (1.80-15.51)
Suicidality		1.17 (0.28-10.39)	1.61 (0.25-10.29)	.15	.28	INT: 7.26 (1.75-30.18) EXT: 2.90 (0.52-16.23)
Mental health concerns[b]	Unhappy /dissatis-faction	5.20 (1.70-15.86)	5.91 (1.88-18.55)	.07	.17	INT: 15.94 (6.43-39.51) EXT: 1.05 (0.36-3.01)
	Poor sleep quality	5.85 (1.26-27.20)	6.12 (1.29-29.06)	.08	.17	INT: 6.11 (2.22-16.86) EXT: 2.63 (0.78-8.89)
	Loneliness	8.06 (0.98-66.37)	9.63 (1.15-80.83)	.13	.22	INT: 17.54 (5.76-53.38) EXT: 1.42 (0.36-5.56)
Serious physical event[a]		2.27 (0.41-12.57)	2.14 (0.38-12.13)	.02	.09	INT: 1.03 (0.20-5.42) EXT: 4.16 (0.75-23.09)
Obesity		1.28 (0.45-3.62)	0.92 (0.29-2.88)	.02	.18	INT: 1.01 (0.29-3.49) EXT: 3.44 (0.93-12.68)
Poor subjective physical health		8.04 (3.02-21.39)	9.37 (3.46-23.38)	.09	.16	INT: 5.92 (2.63-13.31) EXT: 0.70 (0.32-2.15)

Note. OR=odds ratio, 95% CI=95% confidence interval, INT=internalizing, EXT=externalizing, Unadj.=unadjusted model, Adj.=adjusted model (covariates were: childhood functioning, sex, IQ, temperament (effortful control, fear, frustration), family SES, parenting stress, perceived parenting (warmth and rejection), and current mental health).

Covariates printed in *italic* had a protective effect as indexed by an OR<1

[a]adjusted analyses controlled for all covariates except childhood functioning data

[b]adjusted analyses controlled for all covariates except current mental health
