# Supplementary figures and images for: The association of developmental trajectories of adolescent mental health with early-adult functioning
Source: PLoS One. 2020 Jun 10;15(6):e0233648. doi: 10.1371/journal.pone.0233648 (PMC7286481; doi:10.1371/journal.pone.0233648)

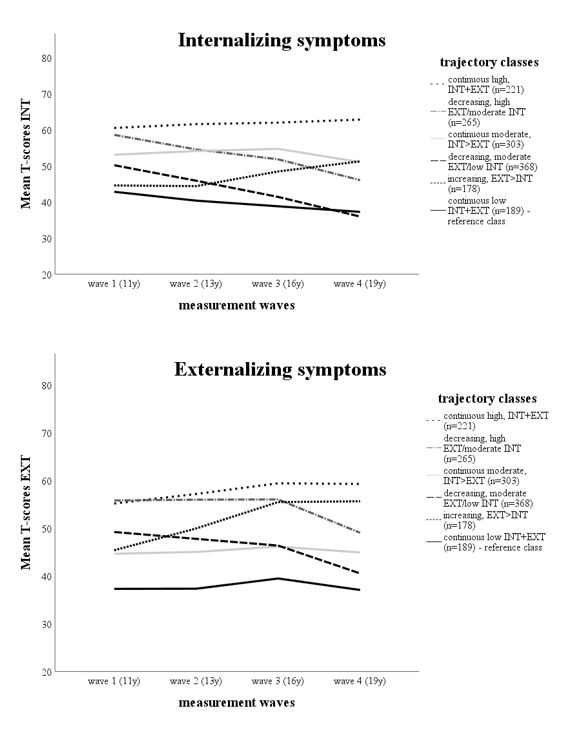

Supplement: S1 Fig — Subclinical and clinical cut-off scores for the YSR/ASR are T = 60 and T≥64 respectively. (TIF) [file pone.0233648.s002.tif]
